# Spatial Pharmacology: Redefining Organelle Contact Sites as Therapeutic Targets

**DOI:** 10.7150/ijbs.129515

**Published:** 2026-01-27

**Authors:** Minjeong Ko, Jiho You, Eunwoo Cho, Ho Jeong Kwon

**Affiliations:** Chemical Genomics Leader Research Laboratory, Department of Biotechnology, College of Life Science and Biotechnology, Yonsei University, Seoul, Republic of Korea.

Inter-organelle communication has emerged as a key regulator of cellular homeostasis and disease pathology 1. Although organelles were once viewed as self-contained compartments, emerging evidence has reframed organelle membrane contact sites (MCSs) as molecularly diverse signaling hubs that coordinate ion exchange, lipid metabolism, and stress responses. Notably, recent studies published in the International Journal of Biological Sciences have contributed to shifting this perspective by highlighting mitochondria-associated membranes (MAMs) as pharmacologically responsive interfaces implicated in neurodegenerative and metabolic disorders 2-4. Chen et al. showed that dietary vanadium exposure remodels the hepatic MAM proteome, disrupting glucose homeostasis and promoting ferroptosis 3, while He et al. demonstrated that omega-3 polyunsaturated fatty acids restore MAM abundance and support spermatogenesis 4. Together, these findings point to a broader conceptual shift in which MCSs are viewed not as passive structural contacts but as dynamic, druggable interfaces, bringing their small-molecule therapeutic potential into clearer focus.

Here, we use the term “**spatial pharmacology**” to describe therapeutic strategies that modulate organelle connectivity rather than individual molecular activities. The implications of this concept extend well beyond the MAM. Recent studies show that diverse organelle contact sites function as key regulatory hubs and can be modulated pharmacologically. In early Alzheimer's disease, Blarcamesine (ANAVEX2-73), an agonist of the Sigma-1 receptor localized at the MAM, demonstrated notable outcomes in clinical trials for Alzheimer's disease (NCT03790709) 5. The flavonoid derivative LW-213 enhanced ER-lysosome interactions through direct binding to lysosomal protein LIMP2, thereby triggering lethal ER stress and suppressing acute myeloid leukemia progression 6. Another natural product, tangeretin, exhibited protective mechanisms against Amyloid beta (Aβ) toxicity by modulating mitochondria-lysosome contacts 7. At the ER-plasma membrane contact sites, the TAT-DP-2 peptide provided neuroprotection in an ischemic stroke model by disrupting the Kv2.1-VAPA interaction 8. Together, these examples illustrate a growing paradigm of “spatial pharmacology,” wherein targeted modulation of organelle connectivity acts as a distinct therapeutic mechanism (**Figure 1**).

MCS-centered pharmacology is emerging as a new direction in drug discovery, connecting systems-level organelle biology with therapeutics ranging from small molecules to peptides. The main challenge now is achieving precise spatial and temporal control so that these strategies can be applied effectively in clinical settings. Because MCSs are structurally complex and highly dynamic, systemic drugs alone may not provide sufficient specificity, and additional approaches will be synergetic for more accurate manipulation of cellular architecture. To this end, emerging technologies such as organelle-targeting nanoparticles and optogenetic tools represent a promising future direction. Nanoparticles can be engineered with specific physicochemical properties to exploit membrane potential or pH gradients, allowing them to enrich payloads at distinct organelle sites 9. This targeted subcellular delivery allows organelle crosstalk to be modulated precisely, in ways that conventional whole-cell drug exposure cannot achieve. Optogenetic/chemogenetic tools that enable graded control of protein associations, such as small-molecule-activated binary association (SAMBA) systems, can also offer the ability to program the timing and duration of contact formation 10. Furthermore, bridging structural understanding with pharmacological intervention will be essential to translate MCS-targeting strategies into clinically actionable therapies.

Overall, these advances make it increasingly possible to control organelle contacts in a precise and predictable way. By adjusting how organelles connect and communicate, we can begin to treat these contact sites not just as structural features of the cell, but as real therapeutic targets. This idea, that cell function can be modulated by adjusting how organelles connect, captures the core of spatial pharmacology and suggests a new direction for future drug development.

## Figures and Tables

**Figure 1:**
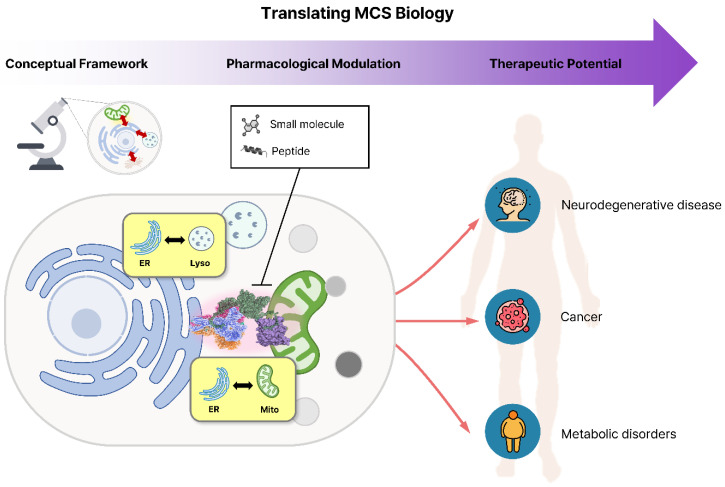
Translating organelle contact site biology into spatial pharmacology and therapeutic intervention. Understanding of organelle membrane contact sites (MCSs) has progressed from structural characterization to therapeutic exploration. Small molecules and peptides that modulate ER-mitochondria or ER-lysosome contact dynamics can reprogram inter-organelle signaling and metabolic coordination, offering new opportunities for therapeutic intervention in neurodegenerative, cancer, and metabolic disorders.

## References

[1] Voeltz G, Sawyer E, Hajnóczky G, Prinz W (2024). Making the connection: How membrane contact sites have changed our view of organelle biology. Cell.

[2] Xue N-J, Liu Y, Lin Z-H, Huang W-H, Zhang F, Zheng R (2026). Parkin deficiency impairs ER-Mitochondria associations and calcium homeostasis via IP3R-Grp75-VDAC1 complex. International Journal of Biological Sciences.

[3] Chen H, Dai X, Xiong Z, Cao H, Xing C, Li H (2026). Dual-pathway mechanism of vanadium-induced hepatotoxicity in ducks: Synergistic crosstalk between glucose homeostasis disruption and NADH/FSP1/COQ10 axis-driven ferroptosis. International Journal of Biological Sciences.

[4] He Z, Ge F, Li C, Zang M, Cao C, Zhang J (2025). The remodeling of Mitochondrial-Endoplasmic reticulum contacts by omega-3 fatty acids mitigates dietary advanced glycation end product-driven Sertoli cell senescence and oligoasthenozoospermia. International Journal of Biological Sciences.

[5] Macfarlane S, Grimmer T, Teo K, O'Brien TJ, Woodward M, Grunfeld J (2025). Blarcamesine for the treatment of Early Alzheimer's Disease: Results from the ANAVEX2-73-AD-004 Phase IIB/III trial. The Journal of Prevention of Alzheimer's Disease.

[6] Zhu My, Guo Yj, Zhu Yq, Wang Hz, Wang Hd, Chen Hy (2025). Activation of lysosomal retrograde transport triggers TPC1-IP3R1 Ca2+ crosstalk at lysosome-ER MCSs leading to lethal depleting of ER calcium. Advanced Science.

[7] He Y, He M-H, Jin T, Wang H-Q, He F (2025). Tangeretin protects against Aβ1-42-induced toxicity and exploring mitochondria-lysosome interactions in HT22 cells. Biochemical and Biophysical Research Communications.

[8] Schulien AJ, Yeh C-Y, Orange BN, Pav OJ, Hopkins MP, Moutal A (2020). Targeted disruption of Kv2. 1-VAPA association provides neuroprotection against ischemic stroke in mice by declustering Kv2. 1 channels. Science advances.

[9] Soukar J, Peppas NA, Gaharwar AK (2025). Organelle-Targeting Nanoparticles. Advanced Science.

[10] Wang T, Liu S, Ke Y, Ali S, Wang R, Hong T (2025). Repurposing salicylic acid as a versatile inducer of proximity. Nature Chemical Biology.

